# Understanding Health Literacy and eHealth Literacy in Nursing Students: A Cross-Sectional Cluster Analysis

**DOI:** 10.3390/nursrep16020052

**Published:** 2026-02-02

**Authors:** Irene Zerilli, Giampiera Bulfone, Donatella Capizzello, Angelo Gambera, Vito Fazzino, Marco Sudano, Antonio Vinci, Fabio Ingravalle, Massimo Maurici

**Affiliations:** 1 Department of Biomedicine and Prevention, Tor Vergata University of Rome, 00133 Rome, Italy; irene.zerilli@students.uniroma2.eu (I.Z.); antonio.vinci@students.uniroma2.eu (A.V.); fabio.ingravalle@students.uniroma2.eu (F.I.); maurici@med.uniroma2.it (M.M.); 2 Department of Medical, Surgical Sciences and Advanced Technologies “GF Ingrassia” (DGFI), University of Catania, 95123 Catania, Italy; donatella.capizzello@asp.sr.it (D.C.); a.gambera@ao-ve.it (A.G.); vito.fazzino@asp.sr.it (V.F.); marco.sudano@unict.it (M.S.); 3 Mediterranean Center of Excellence for the Academic Development of Nursing Research, 80134 Naples, Italy

**Keywords:** health literacy, digital health literacy, eHealth literacy, nursing student, nursing education

## Abstract

**Background:** Health literacy and eHealth literacy are core competencies for nursing students, yet their distribution across training pathways remains insufficiently explored. **Objective:** This study aimed to examine HL and eHL levels among nursing students across different years of the educational programme and identify distinct subgroups of students. **Methods:** A cross-sectional study was conducted among undergraduate nursing students enrolled in all years of a single Italian university programme. Literacy profiles were assessed using validated questionnaires. A Two-Step Cluster Analysis was applied to identify homogeneous literacy profiles. Group differences were examined using appropriate statistical tests. **Results:** Four distinct clusters were identified, showing heterogeneous patterns of literacy profiles across the training course. Significant differences emerged in demographic and educational variables across clusters. **Conclusions:** The findings highlight the coexistence of diverse literacy profiles among nursing students and suggest the need for tailored educational strategies. Due to the cross-sectional design, causal inferences cannot be drawn.

## 1. Introduction

Health literacy (HL) has become a central concept in health promotion and public health. The World Health Organization identifies HL as a fundamental determinant of health and a global public health priority, defining it as the knowledge, motivation, and competencies required to access, understand, appraise, and apply health information in everyday health-related decision-making across the life course [1]. Adequate HL is widely recognized as a prerequisite for equity, empowerment, and sustainability in healthcare systems, and is associated with improved population health outcomes [2]. With the rapid advancement of digital technologies in healthcare, the related concept of electronic health literacy (eHL) has gained increasing relevance. eHL refers to an individual’s ability to access, understand, evaluate, and use health information obtained through digital sources and technologies [3]. While conceptually related to HL, eHL represents a distinct construct, as it additionally requires digital competencies and the ability to critically assess the credibility of online health information. These skills are essential for navigating digital health environments, engaging in self-management, and making informed health decisions in technology-mediated contexts [4].

Evidence consistently indicates that both HL and eHL levels remain suboptimal in many populations, contributing to limited access to health resources, poorer self-management, and increased risks of adverse health outcomes, including complications and hospitalizations [5,6]. Population ageing and the growing burden of chronic diseases further underscore the importance of strengthening HL and eHL to support patient autonomy and shared decision-making [7,8].

In this context, nurses play a key role in promoting both HL and eHL, as they are often responsible for guiding patients through healthcare information, digital tools, and care pathways [9,10]. However, despite their central role, nursing education does not consistently include structured and systematic training focused on the development of HL and eHL competencies, and it remains unclear how these skills evolve throughout nursing education [8,11].

Previous studies have shown that nursing students frequently exhibit insufficient or marginal HL levels [12,13,14,15,16,17,18,19]. Findings on eHL among nursing students are more heterogeneous. Some studies report satisfactory or high eHL levels, particularly among graduate or senior students [8,20,21], suggesting that exposure to academic training and digital resources may support the development of eHL. However, these results are not consistent across studies, and the extent to which HL and eHL follow similar or divergent developmental trajectories during nursing education remains insufficiently understood. From a theoretical perspective, HL may be more closely related to general education, clinical training, and health system exposure, whereas eHL may be more strongly influenced by digital learning environments, technology use, and individual familiarity with online resources. These differences suggest that HL and eHL may not develop uniformly across the training course.

Despite growing interest in this field, empirical evidence remains limited regarding how HL and eHL evolve across different years of nursing education. Moreover, most existing studies have relied on variable-centred approaches, which focus on average trends and may obscure meaningful heterogeneity within student populations. A person-centred approach, such as cluster analysis, allows for the identification of homogeneous subgroups of students with distinct HL and eHL profiles, offering a more nuanced understanding of how these competencies cluster and interact.

Recent studies using cluster-based methods have demonstrated the value of this approach in identifying distinct literacy profiles among university students [22,23]. However, these investigations have largely included students from diverse academic disciplines, and evidence focusing specifically on nursing students remains scarce. Furthermore, little is known about how identified HL/eHL profiles align with different stages of the nursing curriculum, limiting the ability to inform targeted, curriculum-based educational interventions.

In light of these gaps, the present study aims to [1] examine HL and eHL levels among nursing students across different years of the educational programme and [2] identify distinct subgroups of students using cluster analysis.

By adopting a person-centred approach, this study seeks to provide novel insights into the heterogeneity of literacy competencies among nursing students and to inform the development of targeted educational strategies within nursing curricula.

## 2. Materials and Methods

### 2.1. Study Design

The study adopted a cross-sectional design, consistent with the exploratory objectives following the Strengthening the Reporting of Observational Studies in Epidemiology (STROBE) guidelines [24].

### 2.2. Sample and Setting

A convenience sample of nursing students was recruited from a single Nursing Degree Programme in an Italian university in southern Italy. The university enrols approximately 500 new nursing students annually and has a total enrollment of about 1500 students. Participants included students recruited from the 2021/2022 academic year, from all years of the programme who agreed to participate in the study. Students who were out of course (delayed in graduation) were excluded. Sample size was not defined a priori due to this study being exploratory in nature; however, a post hoc sensitivity analysis was conducted using G*Power v. 3.1.9.7 (17 March 2020) for Microsoft Windows to evaluate the sample’s adequacy in satisfying the power level.

### 2.3. Variables and Data Collection

The dependent variables considered are the HL and eHL level, defined, respectively, as “*a fundamental determinant of health and a global public health priority, defining it as the knowledge, motivation, and competencies required to access, understand, appraise, and apply health information in everyday health-related decision-making across the life course*” [1], and as “*an individual’s ability to access, understand, evaluate, and use health information obtained through digital sources and technologies*” [3]. The other investigated variables are sociodemographic, such as employment status (dichotomous: employed vs. not employed), marital status (categorical), age (in years), presence of children (dichotomous), gender (categorical), and academic variables such as year of study (first, second, third), previous attendance to university education (dichotomous), programme choice (first choice in nursing career as decision to pursue nursing as the preferred and initial professional pathway, made in pre-admission test) (first choice vs. not first choice), ECTS credits accumulated during the Nursing Degree Programme (European Credit Transfer and Accumulation System) (continuous), and weighted grade point average (continuous). These variables were considered for the cluster analysis. Their selection was not based on a specific theoretical model; however, it was guided by the previous literature on the determinants of health literacy and electronic health literacy among students [8,12,13,14,15,16,17,18,19,20,21,22,23]. Specifically, the variables were chosen to reflect key domains shown to influence the development of literacy-related competencies, such as academic and sociodemographic characteristics, as identified in the literature on nursing students.

The data collection was carried out during live meetings after university lessons, through a series of structured online surveys. The survey was administered between 1 and 30 April 2024. Two researchers (GB and IZ) explained the aim of the survey. They also provided the students with instructions. During the data collection period, the researchers were available to address any students’ requests for clarification, thereby ensuring consistency and accuracy in data collection.

### 2.4. Measurement

The initial section of the survey gathered sociodemographic information along with data related to the participants’ academic status. In the second part of the survey, we used two instruments: the HLQ [25] to assess HL and the eHL scale [3] to evaluate electronic health literacy.

The HLQ [25] is a 44-item measure that captures the concept of health literacy across nine distinct domains (measured using one scale per domain). The nine scales are as follows:

(1) Feeling understood and supported by healthcare providers;
(2) Having sufficient information to manage my health;
(3) Actively managing my health;
(4) Social support for health;
(5) Appraisal of health information;
(6) Ability to actively engage with healthcare providers;
(7) Navigating the healthcare system;
(8) Ability to find good health information;
(9) Understanding health information enough to know what to do).

Each of the nine scales contains between four and six items that are scored as a graded response. There are four response options for items in the first five scales: strongly disagree, disagree, agree, and strongly agree. Scales 6–9 have a range of five possible responses: cannot do, very difficult, quite difficult, easy, and very easy. Scale scores were devised by summing the item scores and dividing by the number of items in the scale. Scale scores range between one and four for the first five scales, and one and five for scales 6 to 9. Each of the nine scales has been found to be highly reliable (composite reliability ranges from 0.8 to 0.9 for each of the four- to six-item scales) [25]. One-factor confirmatory factor analysis models using Bayesian structural equation modelling [26] confirmed the homogeneity of all scales [27]. In our sample, the Cronbach’s alpha varied from 0.71 to 0.81.

The eHEALS [3] is a 10-item tool composed of two introductory items and eight core items. The first two items assess individuals’ general perceptions of health information available on the internet, while the remaining eight items measure the combined knowledge, comfort, and perceived ability to find, evaluate, and apply electronic health information. Individuals can assess their agreement/disagreement through a five-point Likert scale ranging from “strongly disagree” to “strongly agree”, with a five-point Likert scale (0–4); the total score therefore varied from zero to a maximum of thirty-two, with higher scores representing higher levels of eHL. For the first two items, the Likert scale ranges from “Not useful at all” to “Very Useful” (item 1) and “Not important at all” and “Very important” (Item 2). The eHEALS demonstrated good internal consistency, with a Cronbach’s alpha of 0.86, and an acceptable unidimensional structure, explaining 56% of the total variance [3]. The Italian version of the instrument demonstrated good internal consistency, with a Cronbach’s alpha of 0.86, and an acceptable factorial structure, as previously reported by De Caro et al. [28]. In our sample, the Cronbach’s alpha is 0.90.

### 2.5. Data Analysis

Statistical analyses were performed with SPSS Statistics version 27.0 (IBM Corp., Armonk, NY, USA). Continuous variables were presented as mean, standard deviation, and range. Categorical variables were presented as absolute frequency and percentage.

One-way ANOVA models were used to compare continuous outcome variables (i.e., HLQ and eHEALS scores) across groups defined by academic year and clusters. Categorical variables were compared using chi-square or Fisher’s exact tests, as appropriate.

Prior to ANOVA, data distributions were visually inspected using Q-Q plots. Extreme outliers (4.8%, n = 29) with standardized residuals > ±3 standard deviation (SD) were removed to satisfy normality assumptions and prevent distortion of variance estimates. This procedure slightly reduced the sample size (from 600 to 571) but preserved representativeness. Exclusion of these 29 outliers (few extreme cases about 4.8%) did not meaningfully affect generalizability but strengthened statistical validity by reducing undue influence on mean comparisons.

An exploratory Two-Step Cluster Analysis was conducted to identify heterogeneous student profiles and classify participants into homogeneous subgroups based on the set of mixed variables described above [29].

The selection of clustering variables was guided by previous empirical knowledge about students and health literacy and electronic health literacy, rather than by a predefined theoretical model. Specifically, variables related to academic progression and exposure (year of study, number of ECTS credits earned, weighted Grade Point Average), educational background (previous university attendance and completion of prior university studies), sociodemographic and life circumstances (age, gender, marital status, presence of children, employment status), and educational motivation (first choice) were included. These factors have been shown to influence learning opportunities, access to health information, digital engagement, and the development of literacy-related competencies [8,12,13,14,15,16,17,18,19,20,21].

The log-likelihood distance measure was selected, consistent with standard recommendations for Two-Step Clustering, as it accommodates mixed data distributions (categorical variables treated as discrete probability distributions and continuous variables assumed to follow normal distributions). The optimal number of clusters was automatically determined using the Bayesian Information Criterion (BIC), and the algorithm retained the best-fitting solution. The procedure consisted of two sequential phases: (1) a pre-clustering step that generated small sub-clusters using a sequential clustering approach, followed by (2) hierarchical clustering applied to the pre-clusters to obtain the final cluster solution.

For each cluster, descriptive statistics were computed, including means and standard deviations for continuous variables and absolute/percentage frequencies for categorical variables. Variables contributing most strongly to cluster formation (predictor importance) were identified and reported.

After deriving the final cluster structure, clusters were profiled in relation to HL and eHL level. Group differences across clusters were assessed using one-way analysis of variance (ANOVA) for continuous variables and chi-square tests for categorical variables. When significant main effects were observed, post hoc pairwise comparisons were conducted using Bonferroni correction. Statistical significance was set at *p* < 0.05.

### 2.6. Ethical Issues

This study was carried out in accordance with the principles outlined in the Declaration of Helsinki. The research protocol was approved by the Internal Review Board of the Nursing Degree (*Department Council of 19 October 2022, agenda item No. 43*). Prior to completing the questionnaire, all participants were informed about the objectives of the study. They were assured that their responses would remain anonymous and that all data would be analyzed in aggregate form. Informed consent was obtained from each participant, including their agreement to the publication of the study’s findings.

## 3. Results

### 3.1. Sample Characteristics

A total of 600 nursing students (40.0%) responded to the questionnaire. The mean age of the sample was 22.5 years (±5.08); 75.3% were female, 88.3% were single, and 4.2% had children. Overall, 19.3% were working while attending the programme, and 6.5% of them were employed in a healthcare setting. Regarding academic variables, 65.5% of the nursing students declared nursing as their first choice. All the characteristics are included in Table 1.

### 3.2. Health Literacy Level in Nursing Students

Among the HL dimensions, the highest scores were observed for “Social support for health” (mean 3.11 ± 0.45) and “Appraisal of health information” (mean 3.12 ± 0.38), while the lowest score was observed for “Feeling understood and supported by healthcare professionals” (mean 2.98 ± 0.53). For the remaining dimensions, the highest score was found for “Understanding health information well enough to know what to do” (mean 3.65 ± 0.49) and the lowest for “Navigating the healthcare system” (mean 3.33 ± 0.51) (Table 2).

### 3.3. Differences in Health Literacy Level Among the Year of the Course

Statistically significant differences in HL dimensions were identified across programme years. Specifically, for Dimension 1 (Feeling understood and supported by healthcare professionals), significant differences were observed between first- and second-year students (*p* = 0.002) and between first- and third-year students (*p* = 0.004). In Dimension 3 (Actively managing my health), a significant difference was found between first- and second-year students (*p* < 0.001). Dimension 4 (Social support for health) differed significantly between first- and second-year students and between first- and third-year students (both *p* < 0.001). For Dimension 5 (Appraisal of health information), a significant difference was observed between first- and second-year students (*p* < 0.001). Finally, in Dimension 6 (Ability to actively engage with healthcare professionals), significant differences were found between first- and second-year students (*p* < 0.001) as well as between first- and third-year students (*p* = 0.003) (Table 2).

### 3.4. eHealth Literacy Level in Nursing Students

The verification of the assumptions underlying the ANOVA test required the elimination of outliers (4.8%, n = 29) from an initial sample of 600 students who had response values excessively distant from what was expected. The final statistical analysis was performed on a sample of 571 participants.

The items that show higher scores are “I can tell high-quality health resources from low-quality health resources on the Internet” (mean 2.84 ± 0.77), “I know how to use the Internet to answer my questions about health” (mean 2.79 ± 0.69), and “I know what health resources are available on the Internet” (mean 2.78 ± 0.78). A lower score was shown by “I feel confident in using information from the Internet to make health decisions” (mean 2.08 ± 1.03) (Table 3).

### 3.5. Differences in eHealth Literacy Level Among the Year of the Programme

Statistically significant differences were observed for Item 5 (“I know how to use the health information I find on the Internet to help me”) between first- and second-year students (*p* = 0.035), for Item 6 (“I have the skills I need to evaluate the health resources I find on the Internet”) between first- and second-year students (*p* < 0.001), and for Item 8 (“I feel confident in using information from the Internet to make health decisions”) between first- and second-year students (*p* = 0.012), as well as between first- and third-year students (*p* = 0.019). In addition, a statistically significant difference in the overall score was found between first- and second-year students (*p* = 0.018) (Table 3).

### 3.6. Clusters Definition

We performed a Two-Step Cluster Analysis to identify distinct student profiles based on a combination of sociodemographic and academic variables. Seven students had missing data and were therefore excluded from the Two-Step Cluster Analysis, resulting in a final analytic sample of 564 nursing students. The model’s silhouette coefficient (≈0.3–0.4), classified as ‘Fair’ suggests that the four identified clusters represent genuine structures within the dataset, despite exhibiting moderate separation [30]. This quality level is methodologically consistent with the nature of the data characterized by mixed variables, a relatively homogeneous population, and non-extreme differences between subgroups—as widely reported in educational and health sciences literature. The positive silhouette width confirms that the clusters capture real patterns in students’ academic and personal trajectories; however, the lack of sharp separation reflects the inherent continuity and interdependence characterizing educational pathways.

The results of the cluster analysis show four distinct clusters (Table 4).

Cluster 1—“Second-year students, older in age, with partial active employment.”

This cluster comprises 24.3% of the sample (n = 137). It includes 19.7% (n = 27) of first-year students, 49.6% (n = 68) of second-year students, and 30.7% (n = 42) of third-year students; a small proportion (2.19%, n = 3) had previous university education and all of them completed their prior degree. Nearly half of the students in this cluster (47.4%, n = 65) were employed while attending the programme. Nursing was the first-choice degree for 69.3% (n = 95). The majority were single (81.0%, n = 111), 13.1% (n = 18) had children, and 54.0% (n = 74) were male. The mean age within this cluster was 24.78 years (SD = 7.14). The mean weighted academic grade was 24.62 (±1.92) and the average number of ECTS completed was 63.38 (±31.94).

Cluster 2—“Young second-year students, female and very consistent/regular and without a university background”.

This cluster represents 24.5% of the sample (n = 138). It consists of 5.8% (n = 8) first-year students, 94.2% (n = 130) second-year students, and 0.0% (n = 0) third-year students. All participants in this cluster have no previous university education and are not currently employed. For 59.4% (n = 82), nursing was the first-choice career. Among the students in this cluster, 83.3% (n = 115) are single; the cluster mean age is 20.76 years (±1.54). Additionally, 100% (n = 138) are female and have no children. The mean weighted grade is 25.03 (±1.55), and the mean number of ECTS attended is 58.86 (±16.52).

Cluster 3—“Students with prior education, older in age, and with a pathway not yet completed”.

This cluster encompasses 24.6% of the sample (n = 139). It includes 52.5% (n = 73) first-year students, 37.4% (n = 52) second-year students, and 10.1% (n = 14) third-year students. All participants in this cluster (100%, n = 139) have prior university education, with 17.3% (n = 24) having completed their previous studies. Additionally, 25.9% (n = 36) were employed. Nursing was the first-choice career for 65.5% (91). The mean age of students in this cluster is 24.44 years (±4.90), with 80.6% (n = 112) being female; 2.16 (n = 3) had children, and 90.6% (n = 126) were single. The mean weighted grade is 24.70 (±1.96) and the mean number of ECTS credits attended is 45.72 (±29.73).

Cluster 4—“First-year students without a university background, highly consistent/regular”.

This cluster accounts for 26.6% of the sample (n = 150). It consists solely of first-year students, all of whom have no prior university education and are not employed. Nursing was the first-choice career for 67.3% (n = 101). The mean age of students in this cluster was 20.06 years (±2.24), and 75.3% (n = 113) were female. None of the students in this cluster have children. The mean weighted grade is 24.30 (±1.95) and the mean number of ECTS credits attended is 21.26 (±5.98).

The HL and eHL scores in every dimension are shown in Table 4. Overall, significant differences in health literacy levels were observed across clusters for most dimensions. Cluster 2 showed consistent differences compared with other clusters, particularly in Dimensions 1, 3, 5, 6, and 8, indicating variability in perceived support from healthcare professionals, active health management, appraisal of health information, engagement with healthcare professionals, and ability to find health information. Additional differences were identified between Clusters 1 and 4 in social support for health, and between Clusters 3 and 4 in engagement with healthcare professionals and navigation of the healthcare system. No statistically significant differences were found for Dimension 9, *Understanding health information well enough to know what to do*, or for the overall eHealth literacy level.

## 4. Discussion

This study aimed to assess the levels of HL and eHL among nursing students, examine how these competencies change across the three years of the programme, and identify distinct clusters of students to explore their demographic and educational characteristics. We considered a cross-sectional design. This approach does not allow causal inferences between variables. This is the first Italian study involving nursing students that have identified distinct profiles, which may assist educators in recognizing students’ needs and supporting the development of HL and eHL competencies. In our study, the sociodemographic characteristics of the sample reflect the typical profile of nursing students internationally, as reported in other studies exploring HL and eHL levels among university students [8,31,32]. The findings show that nursing students exhibit varying levels of HL and eHL throughout the programme, and cluster analysis identified four distinct profiles differing in academic stage, prior education, working status, and health literacy patterns, with notable variations in social support, engagement with healthcare professionals, and ability to find health information.

### 4.1. The Health Literacy Level in Nursing Students

The overall HL score indicates a moderate-to-high level of HL across the sample. Mean values for the nine HLQ dimensions ranged from 2.96 to 3.65, suggesting that students generally demonstrate adequate competencies in understanding and managing their health needs. Dimensions related to interaction with the healthcare system, such as the “Ability to actively engage with healthcare professionals” (mean 3.47) and “Ability to find good health information” (mean 3.46), were among the highest-scoring domains. These findings suggest that students perceive substantial interpersonal support and possess satisfactory critical appraisal skills, consistent with previous studies specifically regarding the ability to evaluate and select appropriate health information [17,33,34].

Conversely, “Having sufficient information to manage my health” (mean 2.96) and “Actively managing my health” (mean 2.98) showed comparatively lower, though still adequate, values, indicating potential areas where students may feel less empowered to take autonomous action or may perceive gaps in the information available to them. Similar patterns have been reported by Holt et al. [8] and Balmer et al. [14] in relation to these dimensions, where the score in these two studies is higher. Across the three years of the programme, first-year students consistently reported higher HL levels compared with their senior peers. This pattern may be partly attributable, we hypothesized, to the Italian curriculum structure: early programmes of the first year typically emphasize preventive health, healthy lifestyles, and the identification of reliable health information sources, which may strengthen students’ perceived competencies in several HLQ domains. Furthermore, first-year students often maintain closer ties with family and pre-existing support networks, which may enhance their perceptions of social support, understanding by healthcare professionals, and their ability to actively manage their health. As students progress through the programme and encounter increasing academic and clinical demands, these perceptions may decline, contributing to the lower scores observed in later years.

### 4.2. The eHealth Literacy Level in Nursing Students

In relation to eHL level, items such as “I can tell high-quality health resources from low-quality health resources on the Internet” (mean = 2.84) and “I know what health resources are available on the Internet” (mean = 2.78) were among the highest scoring. These results suggest an adequate capacity to locate and recognize trustworthy digital health information. In contrast, lower scores were observed in competencies related to applying online health information. The items “I feel confident in using information from the Internet to make health decisions” (mean = 2.08) and “I know how to use the health information I find on the Internet to help me” (mean = 2.46) showed the lowest means, highlighting weaker confidence in transforming information into action. These findings suggest that while students possess reasonably developed skills in identifying, locating, and evaluating online health information, they demonstrate less certainty when applying this information to guide health behaviours or decisions. This pattern aligns with findings from previous research showing that younger populations often feel proficient in digital navigation but may lack deeper critical or decision-making skills related to eHL [8,22,35]. There are differences between first- and second-year students in their knowledge of how to use health information, their ability to evaluate health resources, and their capacity to apply this information to decisions about their health. Second-year students showed greater competence in these areas, we hypothesized, likely reflecting the influence of academic exposure and developing critical appraisal skills. These findings highlight the importance of integrating eHL training early into the curriculum to support informed decision-making.

### 4.3. Cluster Analysis

The cluster analysis revealed four distinct student profiles shaped by academic trajectory, age, and employment status. Cluster 1 includes older, partially employed second-year students who maintain satisfactory progress despite extra-academic responsibilities and represent a non-traditional student group. Cluster 2 comprises young, highly regular second-year students with the highest GPA, depicting an ideal and uninterrupted academic path. Cluster 3 consists of older students with prior university experience who show academic maturity but a less linear progression, with credits distributed across the first and second years. Cluster 4 represents young, first-year students without prior university background, showing regular progression and strong academic performance typical of newly enrolled students.

Analysis of HL dimensions across the four clusters revealed clear differences that mirror students’ demographic and academic profiles. While eHL did not vary among groups, several HL dimensions showed significant contrasts. Cluster 4 consistently scored highest in multiple HL dimensions, suggesting greater confidence, autonomy, and engagement with health information. In contrast, Clusters 1 and 3, showed lower scores in several HL dimensions. Cluster 3 displayed challenges in perceiving support from healthcare professionals and appraising health information, while Cluster 1 showed reduced social support and greater difficulty navigating the healthcare system, consistent with the known constraints faced by working adults. Cluster 2, composed of young second-year students, who were female, very consistent/regular, and without a university background, scored lower than Cluster 4, which consists of first-year students who were without a university background, highly consistent/regular, in dimensions, possibly reflecting limited exposure to health systems. Overall, the findings show that HL is strongly influenced by age and academic continuity.

Lima et al. [23], taking into consideration nursing, physiotherapy, nutrition, psychology, and pharmacy courses, also identified a student cluster with the highest HL scores, characterized by a predominance of female students, who were older in age (mean 29.59 years) and enrolled in nursing programmes compared to pharmacy. In contrast, in our study, Cluster 4, similarly achieving the highest HL scores, was predominantly female but notably younger (mean 20.06 years). This difference suggests that while gender may consistently relate to higher HL, age may interact differently depending on the academic context and student population.

#### 4.3.1. Limitations

This study has several limitations that should be considered when interpreting the findings [36]. First, the cross-sectional design precludes the assessment of causal relationships or changes in health literacy and eHealth literacy over time; therefore, developmental trajectories across the nursing curriculum cannot be inferred. Second, the study was conducted within a single university nursing programme, which may limit the generalizability of the results to other educational contexts, institutions, or countries with different curricula and training pathways. Third, data were collected using self-reported questionnaires, which are subject to response bias, including social desirability and recall bias, potentially affecting the accuracy of the measured literacy levels. Additionally, although validated instruments were used, they may not fully capture the complexity of functional, interactive, and critical dimensions of health and eHealth literacy in clinical practice.

Finally, unmeasured factors such as prior exposure to digital health tools, informal [36] learning experiences, or individual motivation may have influenced cluster membership and were not accounted for in the analysis.

#### 4.3.2. Implications for Research

The findings of this study suggest some implications for research. For research, it could be interesting to conduct longitudinal tracking of HL and eHL to analyze changes in health literacy across different academic stages, in order to better understand how student trajectories and life circumstances influence HL development. Other implications are related to the motivation as certain HL and eHL dimensions vary across clusters while others remain stable. Lastly, the four identified student profiles provide a framework for comparing educational outcomes, such as drop-out risks and academic success.

#### 4.3.3. Implication for Education

The first implication relates to the levels of health literacy (HL) and eHealth literacy (eHL) observed among students across different years of the programme. The findings suggest the need to more systematically integrate HL and eHL content and teaching methodologies, particularly during the second and third years of the nursing curriculum. In this regard, it would be beneficial to organize both practical laboratories and theoretical lessons aimed at developing students’ ability to assess HL and eHL levels in different population groups, interpreting literacy-related needs, and adapting communication strategies accordingly. Educational activities should focus on strengthening skills in the use of plain language, shared decision-making, and digital health tools, as well as on critically appraising online health information and supporting patients in navigating digital health resources. The identification of distinct student clusters with differing HL profiles highlights the need for a differentiated and longitudinal approach to the development of HL and eHL within undergraduate nursing education. Rather than adopting a uniform strategy, educational interventions should be aligned with the specific strengths and weaknesses observed across clusters, as identified through the cluster analysis. Students in Cluster 1, who were more advanced in the programme and more frequently combined academic study with employment, demonstrated higher consistency in several HL dimensions but continued variability in domains related to engagement with healthcare professionals and navigation of the healthcare system. Educational strategies for this group should therefore prioritize applied and practice-oriented learning experiences that reinforce these specific competencies. Flexible instructional formats, such as blended learning and case-based activities, may support continued skill development while accommodating external commitments. Simulation-based learning and problem-based scenarios focused on patient communication and system navigation may be particularly relevant, given the HL dimensions in which differences were observed. Cluster 2, composed mainly of younger second-year students without prior university education, showed significantly lower scores across multiple HL dimensions, including appraisal of health information, active health management, and engagement with healthcare professionals. These findings suggest a need for structured and progressive educational approaches aimed at strengthening foundational HL and eHL competencies. Guided theoretical instruction combined with supervised practical activities may support the development of critical skills, particularly in evaluating health information sources and using digital health tools appropriately. Collaborative learning activities may further address the lower confidence levels reflected in this cluster’s HL profile. Students in Cluster 3, who had prior university education but less regular academic progression, exhibited intermediate HL levels, with strengths in certain cognitive domains but differences in dimensions related to healthcare system navigation and professional engagement. Educational strategies for this group should therefore build on existing academic competencies while targeting the specific HL dimensions where gaps were identified. Reflective and analytical learning activities, such as critical appraisal of scientific and digital health resources and project-based assignments, may be effective in consolidating HL skills. Targeted academic mentoring and formative feedback may also help address discontinuities in learning trajectories identified in this cluster. Finally, Cluster 4, comprising first-year students without prior university education but with high academic regularity, showed lower HL levels across several dimensions, consistent with early-stage exposure to higher education and healthcare contexts. For this group, early and scaffolded introduction to HL and eHL concepts is warranted. Introductory lectures, interactive workshops, and basic simulation exercises should focus on developing foundational competencies in accessing, understanding, and applying health information, as well as fostering initial confidence in digital health contexts. Overall, these findings underscore the importance of embedding HL and eHL education across the nursing curriculum using strategies that are explicitly informed by empirically derived cluster profiles. Tailoring educational interventions to cluster-specific HL strengths and limitations may enhance students’ readiness to address diverse health literacy needs in clinical practice and contribute to more effective and equitable patient care.

## 5. Conclusions

This study provides a detailed overview of the HL and eHL levels of Italian nursing students, showing variations across academic years and distinct student profiles. Students generally demonstrated moderate-to-high HL, with strengths in engaging with healthcare professionals and evaluating health information, while autonomous health management and the application of digital information were comparatively weaker. Differences between first- and second-year students suggest that academic progression and exposure to curriculum content enhance critical appraisal and information-use skills, highlighting the importance of integrating HL and eHL training early in the programme.

Cluster analysis identified four profiles influenced by age, academic stage, and employment status, indicating that HL is shaped by both demographic and educational factors, whereas eHL appears more stable. Although previous studies have examined HL and eHL in nursing students, few have explicitly linked these competencies to curriculum structure or employed cluster analysis to define student subgroups. This study offers a novel exploratory perspective by applying cluster analysis to nursing students’ HL and eHL competencies within the academic curriculum. While preliminary, these findings suggest directions for future longitudinal studies and curriculum design improvements.

These findings have important implications for nursing education. Tailored interventions could support students with specific literacy needs, and embedding HL and eHL throughout the curriculum may strengthen competencies essential for informed health decision-making. Future research, especially longitudinal studies, is needed to track changes in HL and eHL over time and to assess the impact of targeted educational strategies.

## Figures and Tables

**Table 1:** Characteristics of the sample.

Sociodemographic Variables		N (%)
Year of the programme	1st year	268 (44.7)
	2nd year	274 (45.7)
	3rd year	58 (9.7)
Gender	Female	452 (75.3)
	Male	148 (24.7)
Age mean (standard deviation, range)	22.5 (5.08, 18–53)	
Marital status	Single	530 (88.3)
	Divorced/separated	4 (0.7)
	Married/cohabiting	65 (10.8)
	Widower	1 (0.2)
Children	No	575 (95.8)
	Yes	25 (4.2)
Number of children	One	12 (2.0)
	Two	12 (2.0)
	Three or more	1 (0.2)
Work during the programme	No	484 (80.7)
	Yes	116 (19.3)
Nursing degree as a first choice	No	207 (34.5)
	Yes	393 (65.5)
Others choice	Physiotherapy	38 (18.4)
	Midwifery	49 (23.6)
	Speech therapist	11 (5.3)
	Medicine	50 (24.2)
	Others	59 (28.5)
Previous university education	No	452 (75.3)
	Yes	148 (24.7)
Weighted mean of grade for overall sample (standard deviation, 95% CI range)	24.7 (1.9, 18–29.1)	
Weighted mean of grade for 1st year of the programme (standard deviation, 95% CI range)	24.1 (2.1, 18.0–28.7)	
Weighted mean of grade for 2nd year of the programme (standard deviation, 95% CI range)	25.1 (1.6, 19.0–29.0)	
Weighted mean of grade for 3rd year of the programme (standard deviation, 95% CI range)	25.4 (1.3, 22.5–29.1)	
ECTS attended mean (standard deviation, 95% CI range) for overall sample	46.9 (28.3, 5–135)	
ECTS attended mean (standard deviation, 95% CI range) 1st year	21.9 (7.2, 5.0–79.0)	
ECTS attended mean (standard deviation, 95% CI range) 2nd year	60.5 (14.6, 10.0–79.0)	
ECTS attended mean (standard deviation, 95% CI range) 3rd year	98.9 (25.4, 27.0–135.0)	

**Table 2:** Health Literacy Questionnaire (25) score in the years of the programme.

	Mean (SD) [95% CI Range]	F *	*p*-Value	1st vs. 2nd Year	1st vs. 3rd Year	2nd vs. 3rd Year
1. Feeling understood and supported by healthcare professionals	2.98 (0.53) [1.00–4.00]	8.51	<0.001	0.002	0.004	0.662
1st year	3.07 (0.54) [1.50–4.00]					
2nd year	2.92 (0.52) [1.00–4.00]					
3rd year	2.83 (0.52) [1.50–4.00]					
2. Having sufficient information to manage my health	2.96 (0.41) [1.75–4.00]	1.93	0.146	0.150	1.000	1.000
1st year	2.99 (0.43) [1.75–4.00]					
2nd year	2.93 (0.40) [1.75–4.00]					
3rd year	2.96 (0.38) [1.75–3.75]					
3. Actively managing my health	2.98 (0.46) [1.40–4.00]	7.31	<0.001	<0.001	0.158	1.000
1st year	3.06 (0.44) [1.40–4.00]					
2nd year	2.92 (0.48) [1.60–4.00]					
3rd year	2.93 (0.35) [2.00–3.80]					
4. Social support for health	3.11 (0.45) [1.40–4.00]	16.37	<0.001	<0.001	<0.001	0.599
1st year	3.22 (0.45) [1.40–4.00]					
2nd year	3.04 (0.45) [1.60–4.00]					
3rd year	2.96 (0.36) [1.60–3.80]					
5. Appraisal of health information	3.12 (0.38) [1.60–4.00]	7.57	<0.001	<0.001	0.214	1.000
1st year	3.19 (0.41) [1.60–4.00]					
2nd year	3.06 (0.36) [1.80–4.00]					
3rd year	3.08 (0.29) [2.00–3.80]					
6. Ability to actively engage with healthcare professionals	3.47 (0.55) [1.40–5.00]	9.57	<0.001	<0.001	0.003	0.894
1st year	3.57 (0.56) [1.80–5.00]					
2nd year	3.40 (0.54) [1.40–5.00]					
3rd year	3.32 (0.51) [2.20–5.00]					
7. Navigating the healthcare system	3.33 (0.51) [1.67–5.00]	2.66	0.071	0.220	0.165	1.000
1st year	3.38 (0.52) [1.67–5.00]					
2nd year	3.30 (0.51) [1.67–5.00]					
3rd year	3.24 (0.46) [2.17–4.67]					
8. Ability to find good health information	3.46 (0.47) [1.80–5.00]	0.62	0.536	1.000	0.973	1.000
1st year	3.48 (0.48) [1.80–5.00]					
2nd year	3.45 (0.47) [2.00–5.00]					
3rd year	3.42 (0.47) [2.40–4.80]					
9. Understand health information enough to know what to do	3.65 (0.49) [1.80–5.00]	0.92	0.398	0.667	1.000	1.000
1st year	3.68 (0.48) [2.40–5.00]					
2nd year	3.63 (0.50) [1.80–5.00]					
3rd year	3.62 (0.48) [3.00–4.80]					

* F = ANOVA test statistics comparing between-group variance to within-group variance.

**Table 3:** eHealth Literacy Scale (3) score in the years of the programme.

	Mean (SD) [95% CI Range]	F * (*p* Value)	1st vs. 2nd Year	1st vs. 3rd Year	2nd vs. 3rd Year
1. I know what health resources are available on the Internet	2.78 (0.78), [0–4]	0.732 (0.482)	0.244	0.514	0.113
1st year	2.74 (0.84), [0–4]				
2nd year	2.82 (0.70), [0–4]				
3rd year	2.81 (0.78), [0–4]				
2. I know where to find helpful health resources on the Internet	2.71 (0.76), [0–4]	0.363 (0.696)	0.395	0.805	0.787
1st year	2.68 (0.85), [0–4]				
2nd year	2.74 (0.65), [0–4]				
3rd year	2.71 (0.86), [0–4]				
3. I know how to find helpful health resources on the Internet	2.73 (0.67), [0–4]	0.319 (0.727)	0.470	0.590	0.922
1st year	2.71 (0.73), [0–4]				
2nd year	2.75 (0.61), [0–4]				
3rd year	2.76 (0.63), [1–4]				
4. I know how to use the Internet to answer my questions about health	2.79 (0.69), [0–4]	0.882 (0.414)	0.185	0.652	0.723
1st year	2.75 (0.74), [1–4]				
2nd year	2.83 (0.62), [0–4]				
3rd year	2.79 (0.69), [1–4]				
5. I know how to use the health information I find on the Internet to help me	2.46 (0.88), [0–4]	2.363 (0.095)	0.035	0.262	0.872
1st year	2.37 (0.93), [0–4]				
2nd year	2.54 (0.84), [0–4]				
3rd year	2.52 (0.80), [0–4]				
6. I have the skills I need to evaluate the health resources I find on the Internet	2.74 (0.76), [0–4]	5.806 (0.003)	< 0.001	0.370	0.242
1st year	2.63 (0.87), [0–4]				
2nd year	2.85 (0.61), [1–4]				
3rd year	2.72 (0.74), [1–4]				
7. I can tell high-quality health resources from low-quality health resources on the Internet	2.84 (0.77), [0–4]	0.552 (0.576)	0.294	0.776	0.724
1st year	2.81 (0.84), [0–4]				
2nd year	2.88 (0.69), [0–4]				
3rd year	2.84 (0.72), [0–4]				
8. I feel confident in using information from the Internet to make health decisions	2.08 (1.03), [0–4]	4.605 (0.010)	0.012	0.019	0.415
1st year	1.94 (1.11), [0–4]				
2nd year	2.17 (0.96), [0–4]				
3rd year	2.29 (0.89), [0–4]				
Overall score	2.64 (0.57), [0–4]	2.969 (0.052)	0.018	0.213	1.000
1st year	2.57 (0.65), [1–4]				
2nd year	2.69 (0.46), [1.50–3.88]				
3rd year	2.68 (0.60), [1.13–4]				

* Fisher test.

**Table 4:** Cluster analysis among nursing students.

Variables	Cluster 1	Cluster 2	Cluster 3	Cluster 4
Nursing students	24.3% (137)	24.5% (138)	24.6% (139)	26.6% (150)
Female	45.9% (63)	100% (138)	80.6% (112)	75.3% (113)
Years of the programme				
1st year	19.7% (27)	5.8% (8)	52.5% (73)	100% (150)
2nd year	49.6% (68)	94.2% (130)	37.4% (52)	-
3rd year	30.7% (42)	-	10.1% (14)	-
Previous university education	2.19% (3)	-	100% (139)	-
Previous university education concluded	2.19% (3)	-	17.3% (24)	-
Work	47.4% (65)	-	25.9% (36)	-
Nursing as a first choice	69.3% (95)	59.4% (82)	65.5% (91)	67.3% (101)
Single	81% (111)	83.3% (115)	90.6% (126)	100% (150)
Children	13.1% (18)	-	2.16% (3)	-
Age	24.78 ± 7.14	20.76 ± 1.54	24.44 ± 4.90	20.06 ± 2.24
Weighted mean grade (±DS)	24.62 ± 1.92	25.03 ± 1.55	24.70 ± 1.96	24.30 ± 1.95
ECTS attended (±DS)	63.38 ± 31.94	58.86 ± 16.52	45.72 ± 29.73	21.26 ± 5.98
HL Dimension 1 (±DS)	2.94 ± 0.58	2.83 ± 0.49	3.05 ± 0.53	3.09 ± 0.48
HL Dimension 2 (±DS)	3.02 ± 0.39	2.87 ± 0.40	2.96 ± 0.39	2.98 ± 0.42
HL Dimension 3 (±DS)	2.98 ± 0.44	2.86 ± 0.45	2.99 ± 0.45	3.08 ± 0.42
HL Dimension 4 (±DS)	3.03 ± 0.42	3.05 ± 0.42	3.12 ± 0.47	3.24 ± 0.42
HL Dimension 5 (±DS)	3.10 ± 0.37	3.01 ± 0.33	3.17 ± 0.38	3.18 ± 0.37
HL Dimension 6 (±DS)	3.53 ± 0.50	3.33 ± 0.51	3.43 ± 0.58	3.63 ± 0.51
HL Dimension 7 (±DS)	3.41 ± 0.48	3.23 ± 0.46	3.26 ± 0.48	3.43 ± 0.50
HL Dimension 8 (±DS)	3.52 ± 0.46	3.35 ± 0.42	3.49 ± 0.45	3.50 ± 0.46
HL Dimension 9 (±DS)	3.70 ± 0.66	3.58 ± 0.42	3.68 ± 0.51	3.66 ± 0.47
eHL total score (±DS)	2.69 ± 0.59	2.59 ± 0.42	2.73 ± 0.57	2.55 ± 0.64

Frequency n (%); means ± standard deviation; HL dimension: health literacy dimension; eHL total score: eHealth literacy total score.

## Data Availability

Data collected in the study are not publicly available due to privacy and ethical restrictions but can be made available upon reasonable request from the corresponding author.

## Public Involvement Statement

Nursing students were involved in this research through responding to the survey. This study was carried out in accordance with the principles outlined in the Declaration of Helsinki. The research protocol was approved by the Internal Review Board of the Nursing Degree (Department Council of 19 October 2022, agenda item No. 43).

## Guidelines and Standards Statement

This manuscript was prepared following the procedures outlined in Strengthening the Reporting of Observational Studies (STROBE). A complete list of reporting guidelines can be accessed via the equator network: https://www.equator-network.org/ (accessed on 17 April 2024).

## Abbreviations

The following abbreviations are used in this manuscript

BIC	Bayesian Information Criterion
ECTS	European Credit Transfer and Accumulation System
eHLS	Electronic Health Literacy Scale
eHL	Electronic Health Literacy
HLQ	Health Literacy Questionnaire
HL	Health Literacy
